# Prevalence and factors associated with regular fast-food consumption among the adult population in Qatar: cross-sectional analysis from Qatar Biobank cohort

**DOI:** 10.3389/fnut.2025.1721023

**Published:** 2026-01-16

**Authors:** Alaa Zuhair Massarweh, Lynne Alexandra Kennedy, Asayel Saleh, Aljazi Al-Thani, Ala Al Rajabi

**Affiliations:** 1 Department of Public Health, College of Health Sciences, QU Health, Qatar University, Doha, Qatar; 2 Clinical Nursing Research, Rumailah Hospital, Hamad Medical Corporation, Doha, Qatar; 3 Department of Nutrition Sciences, College of Health Sciences (QU Health), Qatar University, Doha, Qatar

**Keywords:** Qatar Biobank (QBB), Eastern Mediterranean Region (EMR), fast food consumption (FFC), factors influencing FFC, adult population

## Abstract

**Background:**

The Eastern Mediterranean Region has undergone a rapid nutrition transition over the last three decades, with healthier traditional table diets displaced by energy-dense convenience foods. To the best of our knowledge, this is the first large cohort-based estimate of regular fast-food consumption (RFFC ≥1 time/week) and its correlates among the adult population in Qatar using Qatar Biobank, a volunteer prospective cohort of Qataris and long-term residents.

**Methods:**

A cross-sectional study using a simple randomized sample of 2,000 adult participants from the Qatar Biobank (QBB) longitudinal cohort. Dietary intake was assessed using a validated food-frequency questionnaire. RFFC was modeled as a binary outcome using purposeful multivariable logistic regression with an age^*^sex interaction. A sensitivity analysis on a sub-cohort population to examine the impact of job-related factors.

**Results:**

RFFC was highly prevalent among participants, with 49.7% reporting frequently consuming fast food (≥1 time/week). Age emerged as the strongest independent predictor that was modified by sex (likelihood-ratio test, *p* = 0.034), with the youngest adults (18–24 years) having four-fold higher odds of RFFC compared to the oldest cohort (45–65 years; AORs = 4.36, 95% CI: 2.37–8.02). A significant age–sex interaction was discovered, showing that young women (18–24) had particularly high predicted probabilities of RFFC (78%), which was less than that of their male counterparts by mid-adulthood and reversed slightly in older ages. Low fruit/vegetable intake was significantly associated with RFFC (AOR = 1.30, 95% CI: 1.30–1.60). However, education was positively associated in crude models but lost its significance after adjusting for other covariates and accounting for age–sex interaction. In the sub-cohort population (sensitivity analysis restricted to employed only, *n* = 1,660), night shift schedules were significantly associated with 40% higher odds of RFFC (AOR = 1.40, 95% CI: 1.09–1.81), the age–sex interaction persisted, and low fruit/vegetable intake remained significantly predictive (AOR = 1.37, 95% CI: 1.10–1.71).

**Conclusion:**

RFFC is highly prevalent in this large longitudinal, phenotyped sample of adults residing in Qatar and is concentrated among younger adults, particularly women with low fruit/vegetable intake and among those engaged in night-shift work. These findings highlight the need for age- and sex-specific, as well as occupationally targeted, dietary interventions and food environment strategies to facilitate healthier choices.

## 1 Introduction/Background

Undeniably, there is a marked and global transformation in traditional food consumption and dietary patterns (1–3). Many countries have transitioned away from traditional diets, adopting the so-called “Western diet” (4–6), and highly industrialized food systems instead (7–10), resulting in greater reliance on highly processed convenience fast food (1, 4, 11–15). In response, global fast-food markets have experienced exponential growth (4, 16). For example, in 2021, the US fast food market increased by USD 647.7 billion, and is forecast to grow at an accumulated annual growth rate of 4.6% from 2022 to 2029 (17). Such expansion in fast food markets has been linked to increasingly unhealthy dietary patterns, attributed to diet-related ill health and preventable deaths (1, 10, 16, 18–21).

The pace of nutrition transition occurring in Qatar, like similar oil-rich countries in the GCC (22–26), is, however, unprecedented. Qatar, based in the Middle East, is situated in the largely Arab Eastern Mediterranean Region (EMR), which has a multi-ethnic population of approximately 700 million, living in 22 countries and territories, stretching from Morocco to Pakistan, excluding Algeria (27, 28). The EMR faces a nutritional and epidemiological transition that mirrors global trends, but at a more accelerated pace (27, 29–31). This epidemiological transition, from communicable to non-communicable diseases (NCDs), has occurred rapidly, partly due to the significant increase in dietary risk factors, including increased body weight, body mass index (BMI), blood pressure, blood sugars, and LDL cholesterol (30–32), and the spread of adiposity, related to increased fast food consumption and other diet-related changes associated with greater affluence in the region (28, 33, 34). Indeed, Qatar quickly emerged as one of the wealthiest countries in the world after the discovery of oil and gas in the 1970s (35, 36). Prior to this, Qatari's followed a traditional diet of a limited number of seasonal staple foods (37). This has changed dramatically in a relatively short time period (37). Yet robust estimates from the adult population in Qatar remain scarce; leveraging from QBB allows characterization of regular fast-food consumption (RFFC) patterns in a large, well-phenotyped volunteer cohort.

### 1.1 Fast food consumption (FFC)

Despite the increased and widespread availability of fast food, there is a lack of informative research on the effects of frequent fast-food consumption on health and disease (30, 38–40). Primarily due to the lack of a universally accepted definition of the “frequency” of fast-food consumption (41, 42). Comparison between studies is thus limited also due to the failure to have a standardized definition or standard. For instance, one study might label “frequent” fast food use as ≥2 times per week, while another might define it as ≥1 time per day, making direct comparison challenging (18, 43). Furthermore, inconsistencies in the criterion adopted within or between countries further complicate this (44). It is therefore difficult to estimate the accurate scale of this global problem or to accurately test the potential association.

The definition of fast food also varies. Some refer to fast food as so-called “Western” junk foods, such as burgers, pizza, and fried chips (45–47). In other contexts, traditional items such as shawarma, falafel, and local foods may also be considered (25, 48). In contrast, others define it broadly as the meal that is characterized by ultra-processed food with low nutritional value, readily available and accessible from restaurants (49), while Oxford Dictionary defines fast food as “*food which is kept hot or partially prepared by a snack bar or restaurant, so that it can be served as a quick meal or taken away”* (50).

### 1.2 FFC and health risks crisis

Reports estimate that more than 70% of adults in the GCC region are either overweight or obese, with the slowest pace in Bahrain and the highest in the Kuwaiti population (51). Multiple and interchangeable factors contribute to this alarming trend, including frequent fast food consumption (23, 52–54). Fast food is characteristically calorie dense and nutrient poor; high in sugar, sodium or salt, saturated fat, and low in fiber and refined carbohydrates (12, 13, 45, 46, 55). Evidence suggests FFC with significantly increased body weight and a greater risk of non-communicable diseases (NCDs), including diabetes, cardiovascular diseases (CVDs), and certain cancers (56–62).

Al Jawaldeh et al. showed that approximately 68% of all EMR deaths are related to NCDs (CVD, Cancer, Diabetes, and Stroke), which is more than the global average; obesity, unhealthy diets, and inadequate physical activity are identified as primary drivers of NCD mortality (30, 31, 63). According to a recent Global Burden of Disease Study, diabetes prevalence in Qatar is considered one of the highest in the region, with around 19.9% among individuals aged 20–79, and ranks as the third leading cause of death in the country, with 526 deaths attributed to the disease in 2019 (64). Along the same line, the obesity rate has seen a significant increase of 88% between 2009 and 2019, which indicates the state faces an adiposity crisis, while the salt intake is below average, around 4.21 among both sexes, according to the WHO-instruction level for adults, which is < 5 g per person per day (30, 65).

### 1.3 Factors influencing FFC

Multiple interacting factors have significantly contributed to normalized unhealthy behaviors, mainly driven by the obesogenic environment (66, 67). This environment is shaped by demographic transitions, rapid urbanization, aggressive advertising and marketing, culture favoring convenience and time-saving practices, along with widespread utilization of modern technology, including online delivery platforms (10, 15, 68). Fast food is widely available, accessible, and affordable in Qatar. Several food delivery Applications (e.g., Talabat) exist, and affordable foods, including and especially fast foods, can be ordered and delivered at any time, reflecting the accessibility of fast food (69–71). This pattern has accelerated the prevalence of NCDs in the state due to a continuous increase in overweight/obesity and other metabolic disorders-related behaviors (26, 30, 72–74).

### 1.4 Prevalence of FFC in GCC

The fast-food consumption has become a prominent staple diet in the GCC countries—Saudi Arabia, Kuwait, Bahrain, Oman, Qatar, and the United Arab Emirates—particularly, among youth and young adults (22, 23, 25, 54). The proportion of adults in Qatar who regularly consume fast food is unknown, while 45% of Kuwaiti youth consume fast food at least once a week, followed by 44% in Oman and approximately 39% in the UAE (75). Among young adults, the prevalence is even more complicated, 88.8% in Saudi Arabia (49), nearly 80% in Bahrain (76), and over 90% in some surveys among adults in Kuwait (25).

The trend is slightly lower than in many cases within the EMR. Adolescents in Egypt, Jordan, and Lebanon show weekly fast-food intake rates between 45 and 50% (75). The high prevalence in GCC mirrors trends noted in other high-income countries (HIC), such as in the United States, showed approximately 80% of adults consume fast food regularly (77). Similarly, issues are noted in New Zealand, Malaysia, Australia, and the United Kingdom, reflecting a shared reliance on fast food (23, 77, 78). These patterns, driven by urbanization, high affluence, and cultural shifts, create a gradient in fast food consumption. To date, there has been no all-embracing QBB-Large longitudinal cohort-based analysis of regular fast-food consumption among adults in Qatar.

## 2 Methods and materials

### 2.1 Study design and data source

To assess the prevalence and factors associated with regular fast-food consumption among the adult population, a cross-sectional survey design has been used (79–81). The current study utilizes secondary data from the Qatar Biobank (QBB), a large national volunteer cohort with longitudinal follow-up; detailed methods have been published elsewhere (82, 83).

### 2.2 Participants and study population

A simple random sample of 2,000 eligible participants was drawn from the QBB analytic dataset registry, conditional on eligibility criteria and sex strata, to achieve approximately equal distributions by gender, where every participant had an equal opportunity to be selected if they met eligibility criteria (84, 85). QBB executed sampling according to our prespecified inclusion criteria.

### 2.3 Inclusion and exclusion criteria

The inclusion criteria were (i) completeness of dietary intake data related to fast-food consumption, (ii) age between 18 and 65 years, (iii) an equal male-to-female ratio, and (iv) nationalities represented in the QBB dataset (82). For the exclusion criteria, (i) pregnant or breastfeeding women, (ii) people following restrictive diets, vegetarian diets, Mediterranean diets, or vegan diets, (iii) people aged under 18 years and over 65 years, and (iv) people who had completed QBB surveys between 8 March 2020 and 8 March 2024 to avoid pandemic-era diet shifts and potential impact on dietary habits through allowing a post-restriction washout (86–88). These dates correspond to Qatar's national COVID-19 restriction period and a subsequent 12-month washout in which mobility patterns, food-service availability, and stress-related eating behaviors remained atypical (89, 90). QBB operationalized these eligibility dates during the sampling stage.

### 2.4 Ethical considerations

The study was registered with Qatar Biobank (QBB) under application number QF-QBB-RES-ACC-00294, and ethical clearance was granted from the Institutional Review Board under reference number QF-QBB-RES-ACC-00287. All study procedures were confirmed to the ethical principles of the Declaration of Helsinki (91). All participants provided informed written consent to share their data in research studies without declaring their identity by using a de-identifying number to protect participants' privacy and confidentiality.

### 2.5 Operational definitions and determination

#### 2.5.1 Food frequency questionnaire (FFQ)

Food frequency questionnaires were utilized to assess dietary habitual intake via QBB (83), a validated computer-administered questionnaire designed based on a consultation, field assessments of the local food environment, and focus groups with nutrition researchers (83). FFQ included 102 food items classified into 38 food groups based on nutritional composition and preparation strategies, designed to capture habitual intake of a wide range of food items over the past 12 months (83).

#### 2.5.2 Regular fast-food consumption (RFFC)

For our purpose, the most commonly accepted definition of fast food, according to the English Oxford Living Dictionary 2019, refers to foods cooked partially, kept hot, and served quickly—usually high in fat, sugar, and/or sodium with energy density, ready to eat at restaurants, takeout, or through delivery services (50). Regular fast-food consumption was operationalized as consuming any fast-food item at least once per week, which is compatible with academic scholarly conversation and well-documented literature (15, 45, 52, 55, 92–95).

#### 2.5.3 Body mass index (BMI)

Body mass index (BMI) was estimated using the standard criterion (kg/m^2^) to identify participants into weight categories based on the QBB methodology (83). However, the classification of participants followed the World Health Organization (WHO) criteria (96, 97) and grouped accordingly, underweight (BMI < 18.5 kg/m^2^), normal weight (BMI 18.5–24.9 kg/m^2^), overweight (BMI 25.0–29.9 kg/m^2^), and obese (BMI ≥ 30.0 kg/m^2^).

#### 2.5.4 Leisure physical activity calculation

Leisure physical activity (PA) was identified according to QBB as a categorical variable with four mutually exclusive groups: “no report (no physical activity),” “low,” “moderate,” and “high” derived from participants' self-reported frequency and duration of walking, moderate, and vigorous activity. For Modeling, we followed WHO/epidemiologic practice and dichotomized PA at 600 metabolic equivalent task (MET)-min/week (≥600 vs. < 600), treating the non-reporting (no physical activity) as insufficient exercise by combining it with the < 600 MET-min/week category (98, 99). Total weekly PA was calculated in metabolic equivalent task (MET) minutes by multiplying duration (in minutes) and frequency (days/week) of each activity by its corresponding MET value (100, 101).

#### 2.5.5 Fruit and vegetable intake categorization

Fruit and vegetable intake was categorized according to participants' responses to dietary frequency questions in the QBB database using the median intake scale of consumption, similar to methods used in comparable epidemiological studies (102, 103). Participants with a consumption of ≤ median were classified as having low fruit and vegetable intake, whereas those > median were classified as having high fruit and vegetable intake.

### 2.6 Statistical analysis

All statistical analyses were constructed using Stata (Version 19.5, Stata_Corp LLC, College Station, TX, USA). The quality of the data was assessed for completeness, inconsistencies, and outliers. The statistical significance was set at a two-sided *p*-value < 0.05. Data presented and summarized as frequencies (*n*) and percentages (%) for sociodemographic and health-related characteristics of the study population. Bivariate analysis to evaluate the association between regular fast-food consumption (RFFC) and relevant covariates was conducted using the Pearson χ^2^ test. The prevalence of RFFC was defined as the consumption of fast food ≥1 time per week and was estimated overall and within the strata of key variables.

To identify variables independently associated with RFFC, a multivariable binary logistic regression model was built utilizing a purposeful selection approach for explanatory modeling. The outcome was fast-food consumption (NRFFC: < 1 time/week vs. RFFC: ≥1 time/week). However, a two-stage, purposeful selection analytical approach was adopted. The primary multivariable model was developed for the entire cohort to maximize sample size; however, the complete-case data were used (*n* = 1,993; 99.7% of 2,000) due to isolated missing values (< 0.4% across variables used in the main model; see Supplementary Table S1) on covariates required for logistic regression. This missingness did not influence the results or materially affect representativeness or statistical power. The secondary model (sensitivity analysis) was restructured to include employment-specific variables (those who reported having an income and a job). The sensitivity model is fit within the employed sub-cohort, yielding *n* = 1,660 to assess their independent associations within the working population.

For each model, the univariable logistic regression was performed. Predictors with a univariable *p* < 0.25 or considered relevant clinically and prior literature were included in the initial multivariable model. Non-significant predictors (*p* < 0.05) were dropped one at a time from the model. However, if the estimated coefficients changed by more than 20%, the dropped variable was retained as a confounder. After the model was refitted, variables excluded in the initial step (removed at 0.20) were reintroduced individually to the final model to check for their contribution and significance. Additivity was evaluated, and clinically relevant interactions between key predictors were tested using likelihood-ratio tests. Discrimination was assessed by using the area under the receiver operating characteristic curve (AUC); calibration was done by Hosmer–Lemeshow to evaluate goodness of fit; and an observed-predicted calibration plot was constructed from tenths of predicted risk with a LOESS smooth. Multicollinearity (VIF), model specification (link test), threshold-dependent classification metrics at 0.50, and influence/residual diagnostics were assessed. We did not apply survey weights or calibration to census margins; all estimates reflect the QBB prospective longitudinal volunteer cohort of Qataris and long-term residents.

## 3 Results

### 3.1 Sociodemographic and anthropometric characteristics of the study participants

Of 2,000 participants from QBB, balanced by gender (1,000 men and 1,000 women). The mean age of participants was 39.5 years. The age distribution varied by sex (*p* < 0.001), with the majority of women being older (36.9% aged 45–65 vs. 29.5% in men), whereas men were younger (62.8% aged 25–44 vs. 47.8% in women). Most participants were Qatari nationals (83.8%), but women showed a higher proportion (92.0%) compared to men (75.6%) at *p* < 0.001. Area of residence (urban vs. rural) was balanced across sexes and showed no significant differences. Most participants were well-educated, with 44.5% holding a university degree and 6.1% holding a postgraduate degree, while only 7.4% had primary education or less. Educational attainment significantly differed by sex (*p* < 0.001), where men more frequently reported a bachelor's (45.9%) or postgraduate degree (7.8%) or had technical/professional training. In contrast, women were more likely to have primary education (11.0%) and high-school diplomas (32.1%).

Most participants were in paid employment (65.6%), but there was a notable gender gap (83.4% of men vs. 47.8% of women), with 21.8% of women identifying as housewives. Similarly, night-shift work differed by sex (*p* < 0.001); 48.6% of men reported night-shift work (15.6% working fewer than 2 nights per month and 33.05% working up to three nights per month), whereas only 8.1% of women reported doing so. Moreover, around one-third of participants reported monthly earnings of 20,001–50,000 QAR, with approximately 15.65% reporting earnings of >50,000 QAR per month. However, men were over two times as likely as women to report incomes >50,000 QAR (21.8 vs. 9.3%; *p* < 0.001).

The BMI categories showed that most of the overall participants were obese and overweight (41.5 and 35.7%, respectively), with women showing a higher obesity rate (44.8%) than men (38.2%). In contrast, men had a higher prevalence of overweight (40.6 vs. 30.8%; *p* < 0.001). Smoking behavior showed significant sex disparities (*p* < 0.001); only 9.6% of women had ever smoked, so the rest of them were never smokers, while about 36.5% of men were never smokers, and a high proportion reported daily or occasional, or former smoking (22.5%, 13.5%, and 27.4%, respectively). Noted in the bariatric surgery history, approximately 13.85% of participants had undergone surgery, with a higher rate among women (17.7%) than among men (9.9%; *p* < 0.001). One-third of participants reported no leisure activities (40.0% women vs. 29.2% men). In contrast, among those who reported activity, men were more likely to engage in high-intensity activity (>12,000 MET min/week; 40.4% of men vs. 27.2% of women).

Fast food consumption frequency did not differ significantly between both sexes (*p* = 0.87). However, approximately one quarter of participants (25.4%) ate fast food less than once per week, whereas 26.9% ate fast food once or twice per week, 16.4% ate it three to five times per week, and 6.4% ate it daily, which showed that 49.7% of all participants regularly consumed fast food. In contrast, 25.1% of participants reported never or rarely eating fast food. Finally, 64.6% of men and 52.9% of women reported low fruit and vegetable intake, showing that men did not meet the median consumption threshold (Table 1).

**Table 1:** Sociodemographic and health-related characteristics of the study population by gender (*N* = 2,000).

**Characteristic**	**Male *N* = 1,000**	**Female *N* = 1,000**	**Total *N* = 2,000**	***P*-value**
**Age (years)**				<0.001
18–24	77 (7.7%)	153 (15.3%)	230 (11.5%)	
25–34	310 (31.0%)	234 (23.4%)	544 (27.2%)	
35–44	318 (31.8%)	244 (24.4%)	562 (28.1%)	
45–65	295 (29.5%)	369 (36.9%)	664 (33.2%)	
**Nationality**				<0.001
Qatari	756 (75.6%)	920 (92.0%)	1,676 (83.8%)	
Arab	217 (21.7%)	67 (6.7%)	284 (14.2%)	
Other	27 (2.7%)	13 (1.3%)	40 (2.0%)	
**Area category** ^a^				0.96
Rural	501 (50.1%)	500 (50.0%)	1,001 (50.0%)	
Urban	499 (49.9%)	500 (50.0%)	999 (50.0%)	
**Education level** ^b^				<0.001
Primary school and less	38 (3.8%)	110 (11.0%)	148 (7.4%)	
Secondary school	55 (5.5%)	56 (5.6%)	111 (5.5%)	
High school	280 (28.0%)	321 (32.1%)	601 (30.0%)	
Technical/professional	89 (8.9%)	38 (3.8%)	127 (6.3%)	
University degree	459 (45.9%)	431 (43.1)	890 (44.5%)	
Postgraduate degree	78 (7.8%)	44 (4.4%)	122 (6.1%)	
**Employees status**				<0.001
In paid employment	834 (83.4%)	478 (47.8%)	1,312 (65.6%)	
Retired	42 (4.2%)	84 (8.4%)	126 (6.3%)	
Student/trainee	35 (3.5%)	128 (12.8%)	163 (8.2%)	
Self-employed	11 (1.1%)	2 (0.2%)	13 (0.7%)	
Business/company owner	46 (4.6%)	6 (0.6%)	52 (2.6%)	
Voluntary work	3 (0.3%)	10 (1.0%)	13 (0.7%)	
Unemployed	18 (1.8%)	23 (2.3%)	41 (2.1%)	
Never worked before	10 (1.0%)	51 (5.1%)	61 (3.0%)	
Housewife^c^	0 (0.0%)	218 (21.8%)	218 (10.9%)	
**Monthly income** ^d^				<0.001
< 10,000 or no income	124 (12.4%)	272 (27.2%)	396 (19.8%)	
10,000–20,000	225 (22.5%)	242 (24.2%)	467 (23.4%)	
20,001–50,000	388 (38.8%)	310 (31.0%)	698 (34.9%)	
>50,000	218 (21.8%)	93 (9.3%)	311 (15.6%)	
Do not know	44 (4.4%)	83 (8.3%)	127 (6.4%)	
**Night shift work** ^e^				<0.001
No, never worked at night	514 (51.4%)	919 (91.9%)	1,433 (71.7%)	
Yes, < 2 nights/month	156 (15.6%)	27 (2.7%)	183 (9.2%)	
Yes, ≥3 nights/month	330 (33.0%)	54 (5.4%)	384 (19.2%)	
**BMI categories** ^f^				<0.001
Underweight (< 18.5)	13 (1.3%)	27 (2.7%)	40 (2.0%)	
Normal (≥18.5– < 25)	195 (19.5%)	216 (21.6%)	412 (20.6%)	
Overweight (≥25– < 30)	406 (40.6%)	308 (30.8%)	715 (35.8%)	
Obese (≥30)	382 (38.2%)	448 (44.8%)	830 (41.5%)	
**General health**				0.001
Excellent	357 (35.7%)	279 (27.9%)	636 (31.8%)	
Good	521 (52.1%)	572 (57.2%)	1,093 (54.7%)	
Fair	108 (10.8%)	129 (12.9%)	237 (11.9%)	
Poor	12 (1.2%)	20 (2.0%)	32 (1.6%)	
**Smoking status** ^i^				<0.001
Never smoker	365 (36.5%)	904 (90.4%)	1,269 (63.5%)	
Daily smoker	225 (22.5%)	18 (1.8%)	243 (12.2%)	
Occasional smoker	135 (13.5%)	24 (2.4%)	159 (8.0%)	
Former smoker	274 (27.4%)	54 (5.4%)	328 (16.5%)	
**Bariatric surgery** ^g^				<0.001
No	899 (89.9%)	823 (82.3%)	1,722 (86.1%)	
Yes	99 (9.9%)	177 (17.7%)	276 (13.8%)	
**Fast food consumption**				0.87
Less than once/week	248 (24.8%)	259 (25.9%)	507 (25.4%)	
Once or twice/week	267 (26.7%)	271 (27.1%)	538 (26.9%)	
3–5 times/week	168 (16.8%)	159 (15.9%)	327 (16.4%)	
Every day or almost daily	60 (6.0%)	67 (6.7%)	127 (6.4%)	
Never or rarely	257 (25.7%)	244 (24.4%)	501 (25.1%)	
**Fruit and vegetables intake** ^j^				<0.001
Low fruit and vegetable intake	645 (64.5%)	529 (52.9%)	1,174 (58.7%)	
High fruit and vegetable intake	355 (35.5%)	471 (47.1%)	826 (41.3%)	
**Marital status**				<0.001
Divorced	16 (1.6%)	70 (7.0%)	86 (4.3%)	
Married	825 (82.5%)	560 (56.0%)	1,385 (69.3%)	
Separated	0 (0.0%)	2 (0.2%)	2 (0.1%)	
Single	158 (15.8%)	312 (31.2%)	470 (23.5%)	
Widow	1 (0.1%)	56 (5.6%)	57 (2.9%)	
**Physical activity** ^h^				<0.001
No report	292 (29.2%)	400 (40.0%)	692 (34.6%)	
Low (< 600)	161 (16.1%)	170 (17.0%)	331 (16.6%)	
Moderate (600–12,000)	142 (14.2%)	158 (15.8%)	300 (15.0%)	
High (>12,000)	404 (40.4%)	272 (27.2%)	676 (33.8%)	

The analytic sample is a simple random draw from the QBB registry with a 1:1 sex balance; no nationality quotas were applied. “No report of leisure PA” reflects zero reported minutes of leisure PA, not item non-response. For modeling, PA was dichotomized at 600 MET-min/week (≥600 vs. < 600), with “No report” grouped in the < 600 category. QBB is a volunteer cohort; estimates are QBB-based rather than nationally representative.

Data presented as n (%) for listed variables. *P*-values derived from Chi-square tests.

BMI, body mass index; HDL, high-density lipoprotein; LDL, low-density lipoprotein; MET, metabolic equivalent task; QAR, Qatari Riyals; SD, standard deviation.

^a^Based on area zone the urban from 1 to 69 while rural 70–98.

^b^(less than university degree).

^c^For women (female only).

^d^Both who did not have income as well those who reported income < 10,000.

^e^There are many missing variables because a lot of participants do not have a job (missing because the people who categorized as unemployed did not have a work shift).

^f^Underweight (< 18.5), Normal weight (≥18.5 and < 25.0), Overweight (≥25.0 and < 30.0), Obese (≥30.0).

^g^No: indicate that participants did not have a bariatric surgery where yes: indicate that participants had a bariatric surgery.

^h^Participants were categorized into no leisure time physical activity (0 total MET- minutes/week), low physical activity (< 600 total MET- minutes/week), moderate physical activity (≥600 and < 1,200 total MET- minutes/week), and high physical activity (≥1,200 total MET- minutes/week). (MET minute/week).

^i^Participants were categorized into the following groups: daily smoker: yes, on most or all days, occasional smoker: yes, only occasionally, former smoker: no, stopped smoking or no, have tried smoking once or twice, never smoker: no, have never smoked.

^j^Median intake of fruit and vegetables was used as a cutoff point between high vs. low intake.

### 3.2 Bivariate analysis of regular fast-food prevalence and associated factors

Regular fast-food consumption for this study was operationalized as consuming any fast-food items (Western junk foods such as burgers and pizza or traditional items such as shawarma and falafel) ≥1 time/week. Out of 2,000 adult participants included in this analysis (Table 2), the weekly prevalence of fast-food intake (regular vs. irregular consumers) was stratified by sociodemographic, lifestyle, and clinical determinants using χ^2^ tests.

**Table 2:** Bivariate analysis of sociodemographic, lifestyle, and clinical factors associated with regular fast-food consumption among adults in Qatar (*N* = 2,000).

**Variable**	**Irregular fast-food^a^ consumption (< 1 time/week) *n* (%)**	**Regular fast food consumption (≥1 time/week)** ***n* (%)**	**Total, *N* = 2,000 *n* (%)**	***P*-value**
**Age (years)**				0.000
18–24	57 (24.8%)	173 (75.2%)	230 (100%)	
25–34	165 (30.3%)	379 (69.7%)	544 (100%)	
35–44	274 (48.8%)	288 (51.2%)	562 (100%)	
45–65	512 (77.1%)	152 (22.9%)	664 (100%)	
**Gender**				0.929
Male	505 (50.5%)	495 (49.5%)	1,000 (100%)	
Female	503 (50.3%)	497 (49.7%)	1,000 (100%)	
**Nationality**				0.057
Qatari	829 (49.5%)	847 (50.5%)	1,676 (100%)	
Non-Qatari^b^	179 (55.2%)	145 (44.8%)	324 (100%)	
**Area of residence** ^c^				0.263
Rural	492 (49.2%)	509 (50.8%)	1,001 (100%)	
Urban	516 (51.7%)	483 (48.3%)	999 (100%)	
**Marital status** ^k^				0.000
Single	239 (38.9%)	376 (61.1%)	615 (100%)	
Married	769 (55.5%)	616 (44.5%)	1,385 (100%)	
**Bariatric surgery**				0.013
No	887 (51.5%)	835 (48.5%)	1,722 (100%)	
Yes	120 (43.5%)	156 (56.5%)	276 (100%)	
**Education level** ^d^				0.000
Less than high school	185 (71.4%)	74 (28.6%)	259 (100%)	
High school and higher	823 (47.3%)	917 (52.7%)	1,740 (100%)	
**Employment status** ^e^				0.002
Unemployed	345 (55.5%)	277 (44.5%)	622 (100%)	
Employed	663 (48.1%)	714 (51.9%)	1,377 (100%)	
**Income range (per month)** ^f^				0.000
Low income (< 20,000)	449 (52.0%)	414 (48.0%)	863 (100%)	
Income (20,00–50,000)	317 (45.4%)	381 (54.6%)	698 (100%)	
High income (>50,000)	181 (58.2%)	130 (41.8%)	311 (100%)	
**Night shift work**				0.008
No, never	602 (50.5%)	589 (49.5%)	1,191 (100%)	
Yes, night shift	248 (43.7%)	319 (56.3%)	567 (100%)	
**Body mass index (BMI)** ^g^				0.000
<25 kg/m^2^	166 (36.7%)	286 (63.3%)	451 (100%)	
>25 kg/m^2^	841 (54.4%)	704 (45.6%)	1,544 (100%)	
**General health**				0.980
Excellent	325 (51.1%)	311 (48.9%)	636 (100%)	
Good	547 (50.0%)	546 (50.0%)	1,093 (100%)	
Fair/Poor	135 (50.2%)	134 (49.8%)	269 (100%)	
**Smoking status** ^i^				0.080
Never	821 (51.4%)	776 (48.6%)	1,597 (100%)	
Smoker	187 (46.5%)	215 (53.3%)	402 (100%)	
**Low fruit/vegetable intake** ^j^				0.000
Low intake	522 (44.5%)	652 (55.5%)	1,174 (100%)	
High intake	486(58.8%)	340(41.2%)	826 (100%)	
**Leisure physical activity (PA)** ^h^				0.125
Low	533 (52.1%)	490 (47.9%)	1,023 (100%)	
High	475 (48.7%)	501 (51.3%)	976 (100%)	

The analytic sample is a simple random draw from the QBB registry with a 1:1 sex balance; no nationality quotas were applied. “No report of leisure PA” reflects zero reported minutes of leisure PA, not item non-response. For modeling, PA was dichotomized at 600 MET-min/week (≥600 vs. < 600), with “No report” grouped in the < 600 category. QBB is a volunteer cohort; estimates are QBB-based rather than nationally representative.

Data presented as n (%) for listed variables. *P*-Values derived from Chi-square tests for categorical variables.

BMI, body mass index; HDL, high-density lipoprotein; LDL, low-density lipoprotein; MET, metabolic equivalent task; QAR, Qatari Riyals; SD, standard deviation.

Chi-square tests were performed as appropriate.

^a^Consuming fast food ≥1 time per week indicates that the participant is a regular fast-food consumer, based on the following responses: (Once or twice per week, 3–5 times a week, every day or almost every day). Participants who consume fast food less than once per week are categorized as irregular fast-food consumers, based on the following responses: (Never or rarely, less than once per week).

^b^Non-Qatari: including Arabs and other nationalities.

^c^Based on area zone the urban from 1 to 69 while rural 70–98.

^d^Participants were categorized into less than high school level (did not attend school, did not complete primary school, primary school, secondary school), for high school level and higher (high school, Technical/professional school, university degree, and postgraduate degree).

^e^Participants were categorized into Employed (In paid employment, student/trainee, self-employed, business/company owner, voluntary), and Unemployed [Never worked before, housewife (only for female or women), retired].

^f^ < 20,000: include the participants' income (< 20,000 per month and with no income).

^g^ >25 kg/m^2^ BMI [Underweight (< 18.5), Normal weight (≥18.5 and < 25.0)] while >25 kg/m [Overweight (≥25.0– < 30.0), and Obese (≥30.0)].

^h^Participants were categorized into low leisure time physical activity (< 600 total MET- minutes/week), and high leisure time physical activity (≥600 total MET- minutes/week).

^i^Non-smoker (No, have never smoked, no, stopped smoking, no, just have tried once or twice) and smoker (Yes, only occasionally, yes, on most or all days).

^j^Median intake of fruit and vegetable was used as a cutoff point between high vs. low intake.

^k^Participants were categorized into and unmarried (separated, single, and widowed) and married (married).

Age emerged as a strong inverse relationship and RFFC (*p* < 0.001), where RFFC was most common among the youngest adults (75.2% among the 18–24-year-old group) and steadily declined with age, reaching its lowest point at 22.9% of those aged 45–65 years, reflecting that adults are more likely to rely on fast food. Marital status showed as a significant predictor (*p* < 0.001), with single, divorced/widowed individuals illustrating a higher rate of RFFC (61.1%) than their married counterparts (44.5%), indicating that living with a partner reduces reliance on quick food. Well-educated individuals (52.7%, high school or higher) were more likely to rely on fast food than those less educated (28.6%, less than high school education; *p* < 0.001). Similarly, employed participants were more likely to consume fast food frequently compared to those who were unemployed (51.9% vs. 44.5%, *p* < 0.001). However, the correlation with household income revealed a non-linear pattern (*p* < 0.001), where the middle-income group (20,000–50,000 QAR) showed the highest prevalence of regular fast-food consumption (54.65%) than both low- and high-income groups (48.0 and 41.8%, respectively). Furthermore, participants working night shifts were more likely to consume fast food compared to those who never worked nights (56.3% vs. 49.5, *p* = 0.008). On the contrary, no statistically significant associations were found in RFF based on gender (*p* = 0.929), area of residence (*p* = 0.263), and nationality (*p* = 0.057).

As hypothesized earlier, dietary patterns and overall lifestyle were significantly associated, as individuals with low fruit and vegetable intake were shown to be more likely to be regular fast-food consumers than those with high intake (55.5 vs. 41.2%, *p* < 0.001). Interestingly, that showed a reversible correlation between body size and dietary behaviors to fast food consumption (*p* < 0.001), where participants with a BMI < 25 kg/m^2^ (underweight or normal weight) were more likely to rely on fast food (63.3%) than those with a BMI ≥25 kg/m^2^ (45.6%), indicating that overweight and obese individuals may avoid fast food meals. At the end, it is worth noting that no significant associations were reported in RFFC based on leisure physical time (*p* = 0.125), smoking status (*p* = 0.080), or self-reported general health status (*p* = 0.980).

### 3.3 Consecutive logistic regression models of factors associated with regular fast-food consumption

To assess the association among sociodemographic, lifestyle, and clinical factors in RFFC, a series of logistic regression models was created using the complete-case analytic sample (*n* = 1,993; a summary of missingness by variable is reported in Supplementary Table S1). Table 3 shows the results illustrated as crude, adjusted odds ratios, and the final adjusted interaction model with 95% CIs. Interestingly, the univariate logistic regression replicates the bivariate analysis patterns, which assess the crude association of each variable without controlling for potential confounders. Age was the strongest inverse association, where the odds of RFFC among the youngest age group (18–24 years) were 10-fold higher than older adults (45–65 years; OR = 10.22, 95% CI: 7.01–14.50), while those aged 25–34 years and 35–44 years had eight times and four times, respectively. Being single/divorced vs. married was associated with twice the odds of RFFC (OR = 1.96, 95% CI: 1.61–2.38), whereas education presented a good association, specifically with individuals who had a high-school diploma, which was approximately three times the odds of RFFC compared to those who had less than a high school diploma (OR = 2.78, 95% CI: 2.09–3.70). Being employed (OR = 1.34, 95% CI: 1.11–1.62), having a history of bariatric surgery (OR = 1.38, 95% CI: 1.06–1.78), and consuming fruit/vegetables at low levels (OR = 1.78, 95% CI: 1.49–2.13) were correlated with higher odds of RFFC. Furthermore, those with a BMI ≥ 25 kg/m^2^ were inversely correlated with RFFC, indicating that underweight individuals were more likely to consume fast food (OR = 2.04, 95% CI: 1.65–2.54). On the other hand, the rest of the predictors, such as gender, nationality, area of residence, physical activity, and tobacco consumption, were found not to be statistically significant.

**Table 3:** Sequential logistic regression models of variables associated with regular fast-food consumption among study participants in Qatar.

**Characteristic**	**Crude model^a^ (OR [95% CI]) *N* = 2,000**	***P*-value**	**Adjusted model (main effects)^b^ (AOR [95% CI]) *N* = 1,993**	***P*-value**	**Final model interaction^c^ (AOR [95% CI]) *N* = 1,993**	***P*-value**
**Gender**				
Female	1.00 [0.84–1.20]	0.93	1.17 [0.91–1.50]	0.206	0.82 [0.54–1.23]	0.335
Male	-Reference-		-Reference-		-Reference-	
**Age (years)**				
18–24	10.22 [7.01–14.50]	<0.001	8.50 [5.55–13.01]	<0.001	4.36 [2.37–8.02]	<0.001
25–34	7.73 [5.98–10.00]	<0.001	6.30 [4.72–8.39]	<0.001	5.32 [3.68–7.70]	<0.001
35–44	3.54 [2.77–4.52]	<0.001	3.12 [2.40–4.06]	<0.001	2.55 [1.80–3.61]	<0.001
45–65	-Reference-		-Reference-		-Reference-	
**Nationality** ^d^				
Qatari	1.26 [0.99–1.61]	0.57	1.19 [0.90–1.56]	0.204	1.20 [0.92–1.57]	0.183
Non-Qatari	-Reference-		-Reference-		-Reference-	
**Qatar of residence** ^e^				
Rural	1.11 [0.92–1.31]	0.26	0.97 [0.80–1.18]	0.80	0.99 [0.81–1.20]	0.884
Urban	-Reference-		-Reference-		-Reference-	
**BMI (kg/m** ^2^ **)** ^h^				
<25.0 kg/m^2^	2.04 [1.65–2.54]	<0.001	1.13 [0.88–1.46]	0.307	1.13 [0.88–1.45]	0.352
≥25.0 kg/m^2^	-Reference-		-Reference-		-Reference-	
**Marital status** ^l^				
Single	1.96 [1.61–2.38]	<0.001	1.05 [0.81–1.36]	0.691	1.10 [0.85–1.43]	
Married	-Reference-		-Reference-		-Reference-	
**Education level** ^f^				
High school/Higher	2.78 [2.09–3.70]	<0.001	1.42 [1.02–1.97]	0.036	1.37 [0.98–1.90]	0.64
Less than High school	-Reference-		-Reference-		-Reference-	
**Employment status** ^g^				
Employed	1.34 [1.11–1.62]	0.002	1.21 [0.92–1.58]	0.173	1.18 [0.89–1.55]	0.251
Unemployed^i^	-Reference-		-Reference-		-Reference-	
**General health**				
Good	1.04 [0.85–1.26]	0.67	1.11 [0.89–1.38]	0.329	1.12 [0.90–1.39]	0.323
Fair/Poor	1.03 [0.77–1.37]	0.80	1.14 [0.82–1.59]	0.401	1.15 [0.83–1.60]	0.407
Excellent	-Reference-		-Reference-		-Reference-	
**Physical activity** ^i^				
Low activity	0.87 [0.73–1.03]	0.12	1.05 [0.86–1.29]	0.587	1.07 [0.88–1.32]	0.486
High activity	-Reference-		-Reference-		-Reference-	
**Smoking status** ^j^				
Smoker	1.21 [0.97–1.51]	0.08	1.10 [0.85–1.44]	0.448	1.11 [0.86–1.44]	0.426
Non-Smoker	-Reference-		-Reference-		-Reference-	
**Fruit/vegetable consumption** ^k^				
Low intake	1.78 [1.49–2.13]	0.001	1.30 [1.06–1.60]	0.01	1.31 [1.06–1.60]	0.10
High intake	-Reference-		-Reference-		-Reference-	
**Bariatric surgery**				
Yes	1.38 [1.06–1.78]	0.013	1.22 [0.919–1.63]	0.166	1.24 [0.93–1.66]	0.139
No	-Reference-		-Reference-		-Reference-	
**Age (years)** × **Gender Interaction**				
18–24 × Female	—	—	—	—	2.94 [1.42–6.11]	0.004
25–34 × Female					1.38 [0.81–2.35]	0.23
35–44 × Female					1.52 [0.92–2.53]	0.10
45–65 × Female					-Reference-	

The results from a series of logistic regression models (Crude, adjusted main model, and Final interaction complex model) examining the association between regular fast-food consumption (≥1 time/week) and sociodemographic, socioeconomic, and health-related factors. The analytic sample is a simple random draw from the QBB registry with a 1:1 sex balance; no nationality quotas were applied. QBB is a volunteer cohort; estimates are QBB-based rather than nationally representative. The analytic sample for our multivariable models included 1,993 participants, reflecting the exclusion of only seven individuals (< 0.4%) due to isolated missing values on covariates required for logistic regression; however, we used complete-case analysis via Stata (n = 1,993; 99.7% of 2,000 participants).

^a^The crude model (Model 1) shows unadjusted odds ratios (ORs), with 95% CIs from univariable binary logistic regression for each variable individually (N = 2,000).

^b^The adjusted model (Main effects model) illustrates adjusted odds ratios (ORs) from a multivariable model controlling for all covariates (N = 1,993; LRx^2^ (19) 376.40; Pseudo R^2^ = 0.136).

^c^Final interaction model shows the complex final superior multivariable model, which presents a significant statistical interaction term for Age × Gender (Likelihood-ratio test, p = 0.034), N = 1,993; LRx^2^ (19) = 385.07; Pseudo R^2^ = 0.139.

Regular fast-food consumption was the dependent variable, and irregular consumption (< 1 time/week) served as the reference outcome.

Statistically significant association set at p < 0.05; OR, odds ratio; AOR, adjusted odds ratio, CI, confidence interval.

^d^Non-Qatari: including Arabs and other nationalities.

^e^Based on area zone, the urban from 1 to 69, while rural 70 to 98.

^f^Participants were categorized into less than high school level (did not attend school, did not complete primary school, primary school, secondary school), for high school level and higher (high school, Technical/professional school, university degree, and postgraduate degree).

^g^Participants were categorized into Employed (In paid employment, student/trainee, self-employed, business/company owner, voluntary) and Unemployed (Never worked before, housewife (only for female or women), retired).

^h^ > 25 kg/m^2^ BMI [Underweight (< 18.5), Normal weight (≥18.5 and < 25.0)] while >25 kg/m [Overweight (≥25.0– < 30.0), and Obese (≥30.0)].

^i^Participants were categorized into low leisure time physical activity (< 600 total MET- minutes/week) and high leisure time physical activity (≥600 total MET- minutes/week).

^j^Non-smoker (No, have never smoked, no, stopped smoking, no, just have tried once or twice) and smoker (Yes, only occasionally, yes, on most or all days).

^k^Median intake of fruit and vegetables was used as a cutoff point between high vs. low intake.

^l^Participants were categorized into unmarried (separated, single, and widowed) and married (married).

A complete-case analysis was performed for multivariable logistic regression, yielding an analytic sample of 1,993 participants (99.7% of the whole cohort; missing data were minimal, with < 0.4% across variables). Age remained the strongest finding after adjusting for all other covariates, with 8.5 times the odds of RFFC among the youngest cohort (18–24 years) compared to the old adult group (45–65 years; 95% CI: 5.55–13.01, *p* < 0.001), and the age groups 25–34 years and 35–44 years had 6.30 times and 3.12 times higher odds, respectively. The educational level remained significantly associated with RFFC, although the magnitude of the association declined after adjustment. Participants with a high school education or more had 42% higher odds of RFFC than those with less than a high school education (AORs = 1.42, 95% CI: 1.02–1.97, *p* = 0.036). Furthermore, low fruit/vegetable intake, as an indicator of broader habit patterns, was independently associated with 30% higher odds of RFFC compared to those in high fruit/vegetable consumption (AOR = 1.30, 95% CI: 1.06–1.60, *p* = 0.010). It is worth noting that in the univariate model, there were several variables significantly associated with RFFC, but the multivariable regression rendered them non-significant (e.g., marital status, employment status, bariatric, and BMI) after controlling for other covariates, while other predictors such as gender, nationality, area of residence, and smoking status were not showing significant association in either the crude or adjusted models.

Further interrogation of the multivariable model was performed to uncover the effect of additivity and clinically relevant interactions. The likelihood-ratio test showed a statistically significant interaction between age and sex, with odds (LR χ^2^ (3) = 8.67; *p* = 0.034; Supplementary Table S2), indicating that the association between age and RFFC is not uniform across the population but is rather significantly changed by gender. Consequently, the final interaction model offers the best and accurate fit to the study population characteristics (Table 3). Upon refining the final interaction model, the findings showed that age remained the strongest independent predictor of RFFC. Compared to the aged group 54–65 years, those aged 18–24 years had approximately a four-fold increase in the odds of RFFC (AOR = 4.36; CI: 2.37–8.02), and those aged 25–34 years and those 35–44 years had five-fold and 2.5-fold increases, respectively, in the odds of RFFC. The independent association between low fruit/vegetable intake remained significant, with a 30% increase in the odds (AOR = 1.30; 95% CI: 1.06–1.60). Nonetheless, the inclusion of the interaction term eliminates the magnitude effect of educational level, which was no longer statistically significant (AOR = 1.37, 95% CI: 0.98–1.90, *p* = 0.64), reflecting that the impact of educational status may be partially mediated by age and gender. The rest of the variables were not significant after adjustment.

Although gender alone was not significant across all series models, the interaction term for “female × 18–24 years” was statistically significant (AOR = 2.94, 95% CI: 1.42–6.11, *p* = 0.004), showing that the impact of age on RFFC differs by sex in the youngest cohort, while interaction among other aged groups were not found significant, indicating that young women were particularly likely to consume fast food regularly. To deconstruct this significant interaction, the adjusted predicted probabilities of RFFC were built for each stratum of age and gender from the fitted model, holding all other covariates at their mean value (Supplementary Table S3 and Figure S1). The predicted probabilities illustrate the nature of the effect of the “Age × Gender” interaction, where the RFFC declines steadily with age in both sexes, but gender differences vary by age group. Women aged 18–24 years have the highest predicted probability of RFFC at 78% (95% CI: 0.697–0.871) compared to 60% (95% CI: 0.473–0.731) for their male counterparts. However, the gender gap narrowed among the age group 25–34 (68 vs. 65%), and the gap entirely diminished in the 35–44 years group (52 vs. 47.5%). The pattern gently reversed in the oldest age groups, with men having a higher predicted probability marginally than women (26 vs. 22%). This interaction uncovers that young adults are at high risk for fast food consumption (age-dependent), particularly among women in this cohort, ensuring the necessity for age-gender-tailor-made interventions to bridle RFFC.

The final interaction model showed good calibration, confirming that it fit the data very well (Hosmer–Lemeshow χ^2^ (8) =0.89, *p* = 0.999; Supplementary Table S4), and acceptable discrimination (AUC = 0.745, 95% CI: 0.72–0.77; Supplementary Figure S3). The calibration plot (Supplementary Figure S2) indicated close agreement between observed and predicted probabilities across deciles. Variance inflation factors (VIFs) showed no statistical evidence of problematic multicollinearity (mean = 1.22; max = 1.62; Supplementary Table S5). The link test supported the appropriate specification and had no significant unmodelled non-linearities (_hatsq *p* = 0.623; Supplementary Table S6). Influence/residual diagnostics showed the bulk of residuals are centered near zero; no single observation unduly affected estimates, confirming that the final interaction model and its conclusion about the predictors of RFFC are robust and not driven by a few influential cases (Supplementary Figure S4).

### 3.4 Sensitivity analysis: subgroup purposeful logistic regression

In this stage, the secondary model (sensitivity analysis) was restricted to a specific cohort of employment-related variables (income, night shift status, *n* = 1,660) that were structurally not applicable to non-employed participants to examine how occupational variables altered the pattern of association observed in the whole cohort.

The crude logistic model uncovered that working night shift was significantly associated with a 31% increase in the odds of RFFC (OR = 1.31, 95% CI: 1.07–1.61), and the participants who reported moderate monthly income earnings (20,001–50,000 QAR) were more likely to consume fast food than low-income workers. In contrast, the high-income group revealed a non-significant inverse relationship. In multivariable logistic regression, age remained a significant determinant of RFFC, with those aged 18–24 years having approximately seven-fold higher odds of RFFC (AOR = 6.97, 95% CI: 4.33–11.23, *p* < 0.001) compared to those aged 45–65 years, whereas those aged 25–34 and 35–44 years had odds ratios of 6.87 and 3.23, respectively, at a *p*-value < 0.001. Low fruit/vegetable intake continued to be significantly associated with a high frequency of fast food (AOR = 1.37, 95% CI: 1.01–1.72). Moreover, working night shifts showed a significant correlation with RFFC (1.41, 95% CI: 1.10–1.82). In opposition to the full primary cohort, being a woman in this sub-cohort was significantly associated with higher odds of RFFC (AOR = 1.34, 95% CI: 1.02–1.78, *p* = 0.039), whereas the rest of the covariates were not statistically associated with RFFC after adjustment.

Further examination of the multivariable model for this specific cohort of participants to identify any relevant interactions, which once again displayed that the relation between age and RFFC varied by sex. The likelihood test comparing two models (with and without interaction) exhibited a statistically significant (LR χ^2^ (3) = 8.65, *p* = 0.034), indicating that the interaction model better fit the data of this sub-cohort, reflecting that the sensitivity analysis well reinforced the findings from the full cohort analysis. The graded significant link to age and the statistically strong interaction between age and gender remained the critical dominant predictors of RFFC, as risk among women was confined to younger age groups (18–24 years), with approximately three times more women than men of the same age being at risk of RFFC (AOR__interaction_ = 3.06, 95% CI: 1.33–7.05, *p* = 0.008), whereas sex differences among other age groups were non-significant and diminished.

Low fruit/vegetable intake remained significantly associated with RFFC among the working population (AOR__interaction_= 1.37, 95% CI: 1.10–1.71, *p* = 0.006). It is worth noting that those who work night shifts were significantly associated with a 40% increase in the odds of RFFC compared to those not working night shifts (AOR__interaction_=1.40, 95% CI: 1.09–1.81, *p* = 0.008), showing that working schedule plays a vital role in dietary choice among workers. However, all other covariates, including monthly income, were not statistically significantly associated with RFFC in this sub-population (Supplementary Table S8).

The overall results of the sensitivity analysis verified the key findings from the full sample. The younger age group (18–25 years) and low fruit/vegetable intake were significant and strong predictors of RFFC. In contrast, the influence of age on sex emerged as powerfully substantial on RFFC, particularly for younger women. The discovery of night shift work and female sex as additional predictors in this sub-cohort (working population) indicates that occupational exposure and gender play a significant role in dietary behavior for this specific subgroup. Nonetheless, the rest of the listed covariates, such as educational status, income, and bariatric surgery association, indicate that many of the socioeconomic and health-related factors in the whole cohort do not carry over into the employed subgroup, which validates our main conclusions while spotlighting the need to consider employment context and gender when shaping tailored interventions to curb FFC.

## 4 Discussion

To the best of our knowledge, this is the first large-scale cohort-based analysis to estimate the prevalence of regular fast-food consumption (RFFC) and its associated sociodemographic and health-related risk profile among adults in Qatar, leveraging a random sample from the QBB prospective cohort of Qataris and long-term residents and testing effect modifications. This study addresses a crucial gap in diet-related risk profiling at both national and regional levels, where most studies are fragmented or inadequately investigated, going beyond many of the traditional studies that focus on adolescents or students who are frequently cited (94, 104–106). Additionally, habitual dietary patterns during the COVID-19 restriction period differed markedly from pre- and post-pandemic conditions, with international reports documenting increased consumption of ultra-processed, energy-dense foods and reduced intake of fresh produce (86, 87). Our design analysis focuses on QBB baseline visits conducted outside the COVID-19 emergency and early recovery period, intended to characterize “steady-state” regular fast-food consumption, avoiding conflation with crisis-related shifts toward snack foods, sweets, and lower fruit/vegetable intake documented during the pandemic time (86).

In line with the global acceleration of the nutrition transition, which is characterized by the displacement of healthy traditional diets with energy-dense, nutrient-poor foods (52, 53, 78). Our findings show that RFFC (≥1 times/week) is highly prevalent, with nearly half of the participants (49.7%) reporting fast-food intake at least once per week and unique, socially constructed disparities by age, sex, education, and dietary behaviors. These variations mirror the global and regional changes in eating habits and highlight the role of obesogenic environments, in particular in rapidly transforming Arab Gulf states, where the urbanization, spread of industrialized food systems, western diets, and aggressive food market coverage facilitate the normalization of fast food (6, 17, 30–32).

Although this figure is substantial, it remains lower than estimates from several other Gulf and high-income settings. For example, in one Saudi survey of young adults, approximately 87% reported consuming fast food at least weekly (49), and a Kuwaiti adults' survey showed that approximately nine in ten respondents consumed fast food at least once per week (25). In the United States, analysis of nationally representative dietary-recall data indicates that roughly one-third to two-fifths of adults consume fast food on a given day, with the highest prevalence among those aged 20–39 years (107). However, this phenomenon is not limited to high-income nations; several students based surveys from low and middle income countries reports high level of fast food exposure (26, 108–110), for example, approximately three-quarters of Syrian university students reporting weekly or more frequent intake (111), and approximately two-thirds of Nigerian students consumed fast food daily (108).

No exception to this trend, the RFFC prevalence of our cohort is somewhat higher than the approximately 45%−50% weekly intake reported in some EMR studies (30, 75, 109, 112). Taken together, these surveys suggest that the prevalence of at least-weekly fast food consumption in adults and youth samples typically falls between about 40 and 70%, with higher values observed in selected high-exposure subgroups. Qatar's exceptional economic development and subsequent sociodemographic and agricultural shifts have created an obesogenic environment that makes the picture of FFC accessible and culturally acceptable (36, 53, 66).

Our model revealed the significant inverse relationship between age and RFFC, a pattern consistently documented across settings where fast food intake decreases with age (18, 25, 26, 45, 77, 113), indicating that the youth population is often considered the primary consumers of fast food worldwide and likely reflects cohort effects, generational shifts in food values, or a combination of fast-paced lifestyle and social interactions, limited cooking skills, and increased susceptibility to food marketing among youth (10, 12, 30, 114).

However, the novelty of our finding that the significant association between age and RFFC was modified by gender, where young women at high risk, with almost three-fold higher adjusted odds compared to their male peers—increased adjusted probabilities among youngest women (18–24 years, 78%), convergence by mid-adulthood, and marginal reversal at older ages, adds color to prior regional reports that frequently documented the higher FFC often among men when age is not stratified (15, 25, 92, 108). A recent investigation among Arab adults in Kuwait found that men are more likely to consume fast food than women (25). Similarly, Saudi adult men were found to have a higher proportion of frequent fast-food consumption compared to women (45). This significant interaction underscores the necessity of deconstructing fast-food behavior by both age and gender, particularly in rapidly transitioning societies, where traditional roles, nutritional value, body image norms, cleanliness, and exposure to food environments are changing (25, 78).

Indeed, the higher frequent of FFC among young Qatari women may be related to several mechanisms, such as time scarcity and convenience during education times and early career stages, online app-based delivery that shaving search and cost as young adults, specifically women are quicker adapters to online food delivery (OFD), shifts in sociocultural and social norms around eating behaviors (eating away from home and dislike cooking), role changes and many young women are leading busier lives which may contribute significantly to greater reliance on ready to eat meals (18, 66, 68, 110). These probabilities are closely aligned with findings from other studies showing that higher-educated women are more likely to use fast food, mirroring that social and economic empowerment (15, 95, 115).

According to our study, the relationship between socioeconomic factors and RFFC is a complex and counterintuitive finding that contrasts with numerous existing studies. In the overall cohort, individuals with a high educational level were associated with greater odds of RFFC in both the crude and adjusted models, which is an unexpected discovery that stands out compared to many studies from high-income countries, where lower education is likely to be linked to higher fast-food intake. On the other hand, our findings revealed an intriguing association with income, which was found to be non-linear, with the middle-income group reporting the highest RFFC, suggesting cultural differences may play an important role.

However, a similar trend has been noted in many GCC countries, such as Kuwait (25) and Saudi Arabia (49), where high-level education and income status are paradoxically correlated with RFFC, possibly due to affordability, convenience, social desirability, socializing with friends, and greater marketing exposure (25, 49, 92). Furthermore, this pattern is also mirrored in the U.S. cohort, which found that fast food consumption is not related to the poverty gradient and often peaks in the middle-income distribution, possibly attributed to working hours and time trade-offs (116). Despite this complexity, this association attenuated after the inclusion of age-gender interaction, showing that education may partly play a mediating role in age-related differences.

Nonetheless, these findings diverge from patterns observed in many Western communities, where lower educational attainment and monthly income levels were associated with higher fast food consumption (18), reflecting that the level of education and income status may not influence the frequency and amount of fast food intake. In the sub-cohort group's multivariable model (sensitivity analysis restricted to employee participants only), our findings show that there is no independent relationship between monthly income and RFFC, possibly due to occupational and lifestyle predictors that may conceal the impact of financial resources.

Being single/widowed or divorced was strongly associated with RFFC in the bivariate model, with greater crude odds, indicating that the scale and magnitude of meal patterns in partnered households. However, this association lost its significance once other covariates were controlled, which suggests that marital status is primarily a proxy for life stages, is time-limited, and affordable rather than having an independent influence. This pattern is consistent with many scholarly studies (10, 12, 14, 21, 46, 68). Nationality was found to be non-significant in the overall cohort, which contrasts with the findings among young adults in KSA (45, 47). This difference may be due to the sample cohort being Qatari, unlike the KSA population, which is multinational (49, 117).

It is worth noting that there is an inverse association between BMI and RFFC, where participants with BMI < 25 kg/m^2^ were more likely to consume fast food regularly, which seems counterintuitive, although many scholarly studies supported this association, which was mitigated after adjustment (26, 107, 111). This paradox could be explained by an individual with a high BMI or who is overweight actively avoiding fast food as part of a weight management strategy and changing behavior (reverse causation) after diagnosis, or by underreporting due to social stigma (31, 66, 118–120). Similarly, the history of bariatric surgery was correlated with RFFC in the crude model but not after adjustment, reflecting that post-surgery dietary counseling may eliminate fast-food intake.

The negative correlation between fruit/vegetable and FFC was consistent across all models as an independent predictor. Participants with low fruit/vegetable intake had approximately 30% higher odds of RFFC in the multivariable model. This inverse association has been well-documented across various populations (24, 43, 121), where frequent fast-food consumers often reported lower fruit/vegetable intake and higher refined carbohydrates, high energy density, and sugars or salts (5, 11, 24, 122, 123). This inverse relationship reflects the clustering of unhealthy dietary behaviors that shift from a proper traditional diet to “Westernized” patterns, which is clearly evident in most GCC countries and beyond (30, 46, 49). It echoes the poorer nutritional pattern, which is linked to an increased risk of obesity, metabolic failure, and other non-communicable diseases, such as cardiovascular disorders and some types of cancer (30, 32, 56, 124).

The relationship between employment status and dietary choices is multifaceted and often interacts with socio-occupational related factors and time scarcity, leading to distrust of eating patterns such as eating on the go, a decline in proper family meals (10, 19, 125, 126). Our crude comparison models showed a significant correlation, with employed participants having a greater likelihood of relying on RFFC compared to those unemployed (51.9 vs. 44.5%; OR = 1.34, 95% CI: 1.11–1.62). However, this association lost its statistical significance after adjusting for other covariates, suggesting that employment status is a proxy or mediating factor for main determinants such as age stages (younger workers often consume more fast food), education attainment/income status, and working hours, rather than being an independent predictor. This pattern is consistent across different settings, where individuals in paid jobs shape their dietary choices through time scarcity, long working hours, or irregular schedules, thereby supporting convenience-oriented eating, ready-to-eat meals, or “eating on go” (19, 66, 68, 95, 126–128).

In the sub-cohort model (sensitivity analysis) restricted to participants' related employment factors, working shift and schedule characteristics become salient, where night shift work was significantly associated with approximately 40% higher odds of RFFC. This finding aligns with the increasing body of literature that suggests working shifts has a dose-response relationship based on years of exposure and greater risks in rotating/night schedule, leading to a higher risk of obesogenic conditions and metabolic syndrome (66, 128–131). This mechanism drives this association through the interplay of both socioeconomic and biological factors. For instance, a night shift disrupts the body's natural circadian rhythms, which can negatively affect hormonal regulation, such as leptin and ghrelin, which control appetite and satiety (128, 131). Furthermore, time scarcity and practical constraints of off-hours meal environments limit employees' dietary options, which mainly consist of fast, refined, and processed food that are high in sugar and calories (15, 19, 132, 133). This finding aligns with numerous meta-analysis studies that environments with greater access to fast-food service outlets and limited healthy food choices are associated with higher obesogenic syndrome (44, 129–131, 134).

Interestingly, the age-structure-dependent sex effect persisted in the sub-cohort population (working group), with women aged 18–34 exhibiting three-fold higher odds of RFFC than their male counterparts, suggesting that working women may face specific constraints, such as limited healthy dietary choices on night shift or socio-cultural pressure, resulting in greater fast-food consumption (66, 125, 135). Numerous investigations support the potential sex differences that working women in male-dominated societies may experience regarding time poverty load, hormonal milieu, caregiving, and macro job combination (66, 129, 131). This is a multifaceted interaction of differences in age-gender jointly conditioning fast-food behaviors, calling for a tailored occupational sex intervention such as 24/7 healthier food outlet services and supported home-cooked, more nutritious meals at night shifts through using the substitution effect and strengthening the amount of time available, particularly for female workers who are pressed for time limitations and childcare.

Our findings are situated within a broader obesogenic ecosystem in which accessibility, affordability, proximity, the density of fast-food outlets, online food delivery (OFD) platforms (e.g., Talabat and Snoonu), and extensive marketing, plus genuine or perceived time scarcity and convenience, raise red flags as contributors to poorer food quality, increasing the likelihood of NCD and a potential threat to meeting diet-related Sustainable Development Goals (30, 56, 68, 72, 75, 110, 136).

To effectively address the dramatic increase in FFC observed in Qatar and the wider region, it's crucial to understand the root causes behind adults' dietary choices, especially socio-cultural, which may suggest the decision to consume fast food frequently is often less about a lack of knowledge and more about the interaction among the above predictors. For example, a study conducted in Nigeria found that the main reasons for RFFC were convenience, a wide range of options, and time scarcity/no access to cooking facilities (108). Similarly, a recent US investigation noted a significant association between convenience and disliking cooking, but this correlation did not include a perception of the unhealthiness of fast food (107). However, these public health strategies and policies focused on the negative health effects of fast food may not be effective. Given that, a more promising approach could involve handling the root causes of FFC, particularly among young female workers, by making healthy food more convenient, accessible, and socially appealing.

### 4.1 Implications for policy, practice, and environment

The confluence of our findings across these diverse studies highlights the role of tailored multifaceted interventions, where these results are congruent with Qatar's diet-related NCD profile and the necessity for contextual and segment-specific strategies. Age-sex-tailored interventions focusing on young adults, particularly women, during the life stage of peak RFFC must address not only the health risks of fast food but also the psychological drivers such as convenience, taste, social desirability, peer influence, affordability and time scarcity, and caregiving related to women (137). Workplace-nutrition strategies could offer more appealing, affordable healthy options and policies that regulate night-shift food choices, plus calorie-dense food and portion size may help address occupational drivers of unhealthy eating behaviors (110, 129, 131). Furthermore, our findings showed that low fruit/vegetable intake is one of the strongest predictors of RFFC, suggesting specific programs to increase awareness as well as cultural acceptability and affordability of fresh fruits and vegetables. Policies in the state should move beyond education alone, adapting a systems approach to collaborate with digital platforms such as online food delivery (OFD) that address cultural determinants and modify defaults, promotions, and sorting toward healthier options at peak ordering times (138, 139), plus the display of calorie information on menus as implemented by the Saudi Food and Drug Authority (92).

### 4.2 Strengths and limitations of the study

To the best of our knowledge, this study is the first Qatar Biobank-based analysis of regular fast-food consumption in Qatar. Despite its benefits from a large, well-characterized volunteer cohort with balanced sex representation, robust statistical modeling, interaction testing, and detailed model-performance evaluation (excellent calibration and acceptable discrimination). Nevertheless, QBB is a volunteer cohort drawn from the resident population of Qatar and, although broadly population-based in intent, is not a probability sample and stratified within QBB to balance sex; therefore, our estimates are interpreted as QBB cohort–based. No post-stratification/calibration weights to census margins; as such, selection mechanisms (e.g., healthy-volunteer effects). A cross-sectional design prohibits causal inference; self-reported dietary data are susceptible to recall and social desirability biases; high BMI participants may underreport fast food intake, potentially biasing the BMI association toward null or reversal; the absence of a standardized definition for “regular fast-food consumption” across studies limits direct comparison, though our operationalized definition aligns with scholars' standard practices. Additionally, despite adjustment for a wide range of sociodemographic and lifestyle covariates, residual confounding from unmeasured contextual and structural factors is probable, including household food responsibilities, density and proximity, and broader psychosocial stressors. Finally, by excluding QBB assessments conducted between March 2020 and March 2024, our estimates describe regular fast-food consumption patterns outside the COVID-19 emergency and immediate recovery period; pandemic-related shocks to diet and their interaction with socioeconomic inequalities could not be evaluated and should be addressed in future work using extended QBB extracts that span this interval.

## 5 Conclusion and future directions

The picture of nutritional status in the Eastern Mediterranean Region (EMR) has changed dramatically over the last 30 years, transitioning from traditional diets, often healthier foods, to the consumption of high-energy-dense, low-nutrient diets, “Western foods,” driven by rapid economic growth, urbanization, and food system industrialization.

Accordingly, this prospective longitudinal volunteer cohort of Qataris and long-term residents study provides the first comprehensive estimate of regular fast-food consumption (FFC) among participants and its correlates, revealing a high prevalence of RFFC, with approximately half of the adult population reporting frequently consuming fast food (49.7%). A particularly novel finding is the inverse age gradient, with young adults at high risk and a significant age × sex interaction, with predicted probabilities that were highest among women aged 18–24 years and converged by mid-adulthood. The study also confirms that low fruit/vegetable intake is significantly associated with RFFC.

In contrast, crude differentials by work status attenuated after adjustment, implying mediation through life stages and time structure factors. However, in employee sub-cohort analysis, night shift schedules were significantly associated with about 40% higher odds of RFFC, highlighting the dietary impact of circadian disruption and off-hour food access. These findings corroborate regional evidence that links Westernized dietary patterns to accelerated risk of obesogenic syndrome and NDCs and underscore the need for tailored multi-sectoral interventions that consider socio-economic status, work schedules, and cultural norms. Further steps should move beyond general advice and focus on actionable, culturally specific, evidence-based approaches, such as creating a more supportive food environment, standardized exposure definitions, and prioritizing longitudinal studies to better understand causal relationships.

## Data Availability

The datasets presented in this article are not readily available because data summaries and analyses are presented in the article. The full datasets are available from Qatar Biobank upon reasonable request and subject to Qatar Biobank access procedures. Requests to access the datasets should be directed to Qatar Biobank, https://www.qphi.org.qa/.
