# Supplementary material for: Prevalence and factors associated with regular fast-food consumption among the adult population in Qatar: cross-sectional analysis from Qatar Biobank cohort
Source: Front Nutr. 2026 Jan 16;12:1721023. doi: 10.3389/fnut.2025.1721023 (PMC12855070; doi:10.3389/fnut.2025.1721023)
Supplement: Supplementary file 1 [file Table_1.docx]

**Supplementary Material**

| ***Supplementary Table S1***. Variable-level missingness, modelling treatment, and applicability (*N = 2,000*) | | | | |
| --- | --- | --- | --- | --- |
| Variable  (Analytical coding) | Missing, N | Missing % | How used in modelling | Applicability notes |
| Age (18–24/25–34/35–44/45–65) | 0 | 0.00 | Categorical; reference 45–65 | Main & sensitivity |
| Sex (female/male) | 0 | 0.00 | Binary; reference male | Main & sensitivity |
| Nationality  (Qatari vs non-Qatari) | 0 | 0.00 | Binary | Main & sensitivity |
| Area (rural vs urban) | 0 | 0.00 | Binary | Main & sensitivity |
| Education (≤HS vs >HS) * | 1 | 0.05 | Binary (purposeful selection) | Main & sensitivity |
| Marital status (married vs unmarried) | 0 | 0.00 | Binary | Main & sensitivity |
| BMI (≥25 vs <25) \† | 5 | 0.25 | Binary: 4-level used descriptively | Main & sensitivity |
| General health (Excellent/Good vs Fair/Poor) | 2 | 0.10 | Binary | Main & sensitivity |
| Leisure physical activity (≥600 vs <600) \‡ | 1 | 0.05 | Dichotomized at 600 MET-min/week; “No report” grouped with <600 | Main & sensitivity |
| Smoking (smoker vs non-smoker) | 1 | 0.05 | Binary | Main & sensitivity |
| Fruit & vegetables (low vs high) | 0 | 0.00 | Binary (median split) | Main & sensitivity |
| Bariatric surgery (yes/no) | 2 | 0.10 | Binary | Main & sensitivity |
| Night-shift work (never/ <2/ ≥3 nights/mo.) | **—** | **—** | Not in main model; categorical in sensitivity model | Applicable to employed sub-cohort only (n = 1,660) |
| Monthly income (categorical) | **—** | **—** | Not in main model; categorical in sensitivity model | Applicable to employed sub-cohort only (n = 1,660) |
| Complete-case N (main models) | **—** | **—** | 1,993 (99.7%) | — |
| Sensitivity-model N (employed) | **—** | **—** | 1,660 | Employed sub-cohort |
| ***Footnote. Table S1***shows variable-level missingness, modelling treatment, and applicability across all modelling.  The analytic sample for multivariable models included 1,993 participants, reflecting the exclusion of only seven individuals (<0.4%) due to isolated missing values on covariates required for logistic regression utilized complete-case analysis via Stata (n = 1,993; 99.7% of 2,000 participants).  Employment-specific variables (monthly income; nightshift) are applicability-restricted rather than missing in the main model; their model is fit within the employment-applicable sub-cohort (n = 1,660). “No report of leisure PA” indicates zero reported leisure PA (included in <600 group for modelling).  ***** ≤HS: less than high school; >HS: high school or higher.  **\†** BMI 4-level categories retained in descriptives.  **\‡** “No report” in QBB denotes zero reported leisure PA, not item non-response | | | | |

| **Supplementary Table S2.** Likelihood-Ratio Test Comparing Nested Logistic Regression Models (age×sex interaction) | | | | |
| --- | --- | --- | --- | --- |
| Model Specification | Log-Likelihood | Degrees of Freedom (df) | Likelihood-Ratio (LR) χ² | p-value |
| Model 1: Main Effects Only | -1193.17 | 16 | - | - |
| Model 2: Main Effects + (Age x Gender Interaction) | -1188.84 | 19 | 8.67 | 0.034 |
| ***Footnote. Table S3.*** shows the likelihood test ratio that compares the two nested models.  Model 1 is the final adjusted model (multivariable model), with only the main effects, derived from the purposeful selection approach.  Model 2 adds the three-degree-of-freedom interaction term for age and gender (Final multivariable interaction model).  The significant p-value (<0.05) indicates that model 2 provides a statistically significant improvement and a better fit of the data in the model over model 1, which justifies the inclusion of the interaction term in the final model. The LR chi^2^ value likelihood-ratio test, calculated based on the difference in the log likelihoods of the compared models. Rob > chi2 represents the p-value associated with the test statistic. | | | | |

| ***Supplementary Table S*3. Adjusted Predicted Probabilities of Regular Fast-Food Consumption by Age and Gender** | | |
| --- | --- | --- |
| **Age group** | **Male pr (95 % CI)** | **Female pr (95 % CI)** |
| **18–24 yr** | 0.602 (0.473–0.731) | 0.784 (0.697–0.871) |
| **25–34 yr** | 0.649 (0.568–0.730) | 0.675 (0.590–0.760) |
| **35–44 yr** | 0.469 (0.381–0.557) | 0.523 (0.431–0.616) |
| **45–65 yr** | 0.258 (0.185–0.330) | - 1. (0.158–0.283) |
| ***Footnote. Table 4*** ***shows the significant interaction between age and gender from the final multivariable logistic regression model.***  ¹ Adjusted predicted probabilities, with their 95% CI, were obtained from a binary logistic regression model including the age-gender interaction term, with all other covariates held at their mean values (nationality, area of residence, education level, employment status, body‑mass index category, self‑rated health, physical activity, smoking status, fruit and vegetable intake, marital status, and bariatric surgery history (n = 1 993).  Regular fast‑food consumption is most prevalent among younger adults, declining steadily with age, and the gender gap is pronounced only in the youngest cohort. After the age of 25, men and women show broadly similar patterns, suggesting that interventions to reduce fast‑food consumption may need to be tailored differently for young women than for other demographic groups. | | |

**Supplementary Figure S1. Adjusted Predicted Probability of Regular Fast-Food Consumption, by Age Group and Gender**

***Footnote. Figure S1.*** illustrates a post-hoc analysis of the significant interaction between age and gender that was derived from the final logistic regression model. *The y-axis* ranges from 0 (a 0% probability) to 1.0 (a 100% probability), *represents the predicted probability of consuming fast food regularly (≥1 time/week). Probabilities and 95% Confidence Intervals are adjusted for all other covariates in the final model;* X-axis: age in years, categorized into four groups, which is the primary predictor you are examining in the interaction. Blue line (male) and Red line (female): These lines connect the adjusted predicted probabilities for males and females across the different age groups. Green Line (as balanced): represents the *average* predicted probability for each age group, averaging across both males and females.

| **Supplementary Table S4.** **Calibration Test** (Hosmer-Lemeshow Goodness-of-Fit Test for Final Interaction Model), N= 1,993. | | | | | | |
| --- | --- | --- | --- | --- | --- | --- |
| Group | Mean Predicted Probability | Observed RFFC (N) | Expected RFFC (N) | Observed Non-RFFC (N) | Expected Non-RFFC (N) | Total (N) |
| 1 | 0.1994 | 37 | 34.7 | 164 | 166.3 | 201 |
| 2 | 0.2405 | 45 | 44 | 155 | 156 | 200 |
| 3 | 0.2945 | 50 | 52.2 | 147 | 144.8 | 197 |
| 4 | 0.4726 | 78 | 79.1 | 122 | 120.9 | 200 |
| 5 | 0.5304 | 97 | 99.7 | 102 | 99.3 | 199 |
| 6 | 0.5944 | 109 | 111.1 | 90 | 87.9 | 199 |
| 7 | 0.664 | 130 | 126.7 | 70 | 73.3 | 200 |
| 8 | 0.706 | 138 | 138.1 | 63 | 62.9 | 201 |
| 9 | 0.7567 | 148 | 146.5 | 53 | 54.5 | 201 |
| 10 | 0.8572 | 156 | 156 | 39 | 39 | 195 |
| Overall |  | 988 | 988.1 | 1005 | 1004.9 | 1,993 |
| ***Footnote. Table S4*** shows the Hosmer-Lemeshow Goodness-of-Fit Test for Final Model. Hosmer–Lemeshow χ²( 8)=0.89, p=0.999. RFFC, Regular Fast-Food Consumption. | | | | | | |

**Supplementary Figure S2.** Calibration plot for the final logistic model: observed vs predicted probability of RFFC by deciles, with LOESS smoother and 45° reference line.

***Footnote. Figure S2.*** Points are observed event rates within tenths of predicted risk (size weighted by decile n); the solid curve is a LOESS fit; the 45° line indicates perfect calibration. Hosmer–Lemeshow χ²(8)=0.89, p=0.999 (see Supplementary Table S4).

| **Supplementary Table S5.** Multicollinearity Diagnostics – Final Interaction Model. | | |
| --- | --- | --- |
| Variable | VIF | 1/VIF (Tolerance) |
| Age Category | 1.51 | 0.6603 |
| Gender | 1.62 | 0.6192 |
| Nationality | 1.08 | 0.929 |
| Area of Residence | 1.01 | 0.9921 |
| Education Level | 1.21 | 0.8287 |
| Employment Status | 1.38 | 0.724 |
| BMI Category | 1.18 | 0.8495 |
| General Health | 1.06 | 0.9392 |
| Physical Activity | 1.09 | 0.9135 |
| Smoking Status | 1.21 | 0.8249 |
| Fruit & Vegetable Intake | 1.09 | 0.9181 |
| Marital Status | 1.39 | 0.7201 |
| Bariatric Surgery | 1.05 | 0.9538 |
| Mean VIF | 1.22 | ----------------------- |
| **Table S5**shows the multicollinearity diagnostics for the final model. Multicollinearity (VIF), Variance Inflation Factor. A VIF < 5 indicates no significant multicollinearity. | | |

| **Supplementary Table S6**. Specification Error (Link-Test) for the Final Interaction Model | | | | |
| --- | --- | --- | --- | --- |
| Variable | Coefficient | Std. Err. | z | p-value |
| _hat (Linear Prediction) | 1.008 | 0.059 | 17.18 | <0.001 |
| _hatsq (Squared Prediction) | 0.033 | 0.066 | 0.49 | 0.623 |
| ***Footnote. Table S6***. Shows the specification error test for the final model. The output shows two predictors: _hat (the predicted value, p<0.005) and _hatsq (the predicted value squared, p>0.005). A non-significant p-value for _hatsq indicates the model is well-specified. | | | | |

| **Supplementary Table S7.** Classification performance at decision threshold 0.50 for the Final Interaction Model Predicting Regular Fast-Food Consumption (RFFC ≥1/week) | | | |
| --- | --- | --- | --- |
| Metric | Estimate | 95% CI | Numerator / Denominator |
| Sensitivity (TPR) | 75.30% | 72.52–77.89% | 744 / 988 |
| Specificity (TNR) | 64.18% | 61.17–67.08% | 645 / 1005 |
| Positive predictive value (PPV) | 67.39% | 64.57–70.09% | 744 / 1104 |
| Negative predictive value (NPV) | 72.55% | 69.53–75.39% | 645 / 889 |
| Accuracy | 69.69% | 67.64–71.67% | 1389 / 1993 |
| **Footnote. Table S7.** TP=true positives; FP=false positives; TN=true negatives; FN=false negatives. Sensitivity=TP/(TP+FN); Specificity=TN/(TN+FP); PPV=TP/(TP+FP); NPV=TN/(TN+FN); Accuracy=(TP+TN)/N. 95% Confidence Intervals are Wilson score intervals. Counts derived from Stata 'estat classification' at probability threshold 0.50 (TP=744, FP=360, FN=244, TN=645; N=1,993). | | | |

**Supplementary Figure S3**. Receiver operating characteristic (ROC) curve for the Final Logistic Model Predicting Regular Fast-Food Consumption (RFFC ≥1/week).

***Footnote. Figure S3.*** The area under the ROC curve (AUC) was 0.745; 95% CI estimated using DeLong’s method. The diagonal line denotes no-discrimination. ROC/AUC summarize threshold-independent discrimination; threshold-specific metrics are reported separately (Supplementary Table S7).

| **Supplementary Figure S4**. Diagnostic Plots for the Final Interaction Model: standardized Pearson and deviance residuals vs predicted probability and observation index; leverage (hat) vs predicted probability and observation index; and Pregibon’s delta-beta vs observation index. | |
| --- | --- |
|  |  |
|  | ** |
| ** |  |
|  |  |
| ***Footnote.***  ***Supplementary Figure S4****.* Diagnostic plots of residuals, leverage and influence — lists a–g, show the standardised Pearson residuals and deviance residuals against predicted probability and observation index, leverage against predicted probability and observation index, and Pregibon’s delta beta against observation index *Dotted horizontal lines indicate zero residuals and that points labelled with anonymised IDs identify potential outliers.* | |

| **Supplementary Table S8.** Sensitivity Analysis (Employed Sub-Cohort) Series Logistic Regression Among Employed Participants Related Factors (income and night shift status, n=1,660) | | | | | | |
| --- | --- | --- | --- | --- | --- | --- |
| Characteristic | Crude Model¹ (OR [95% CI])  N = 2,000 | p-value | Adjusted Model (Main Effects) ² (AOR [95% CI])  N = 1,660 | p-value | Final Model Interaction ³ (AOR [95% CI])  N = 1,660 | p-value |
| **Gender**  Female  Male | 1.00 [0.84 – 1.20]  -Reference- | 0.93 | 1.34 [1.01-1.77]  -Reference- | 0.039 | 0.81 [0.50 – 1.29]  -Reference- | 0.373 |
| **Age (years)**  18 – 24  25 –34  35 – 44  45 – 65 | 10.22 [7.01 – 14.50]  7.73 [5.98 – 10.00]  3.54 [2.77 – 4.52]  -Reference- | <0.001  <0.001  <0.001 | 6.79 [4.32 – 11.23]  6.86 [5.03 – 9.36]  3.23 [2.43 – 4.28]  -Reference- | <0.001  <0.001  <0.001 | 3.81 [1.97 – 7.37]  5.37 [3.64 – 7.90]  2.46 [1.71 – 3.54]  -Reference- | <0.001  <0.001  <0.001 |
| **Nationality** ^aa^ |  | 0.57 | 1.13 [0.82– 1.57]  -Reference- | 0.452 | 1.14 [0.82 – 1.59]  -Reference- | 0.402 |
| Qatari  Non-Qatari | 1.26 [0.99 – 1.61] |  |  |  |  |  |
|  | -Reference- |  |  |  |  |  |
| **Qatar of residence** ^bb^ | 1.11 [0.92 – 1.31]  -Reference- | 0.26 | 1.03 [0.84 – 1.28]  -Reference- | 0.727 | 1.05 [0.84 – 1.30]  -Reference- | 0.647 |
| Rural |  |  |  |  |  |  |
| Urban |  |  |  |  |  |  |
| **BMI (Kg/m^2^) ^ee^**  <25.0 Kg/m^2^  ≥25.0 Kg/m^2^ | 2.04 [1.65 – 2.54]  -Reference- | <0.001 | 1.22 [0.93 – 1.61]  -Reference- | 0.139 | 1.21 [092 – 1.59]  -Reference- | 0.165 |
| **Marital status ^ii^**  Single  Married | 1.96 [1.61 – 2.38]  -Reference- | <0.001 | 1.02 [0.77 – 1.36]  -Reference- | 0.876 | 1.05 [0.78 – 1.41]  -Reference- | 0.707 |
| **Education level ^cc^**  High school / Higher  Less than High school | 2.78 [2.09 – 3.70]  -Reference- | <0.001 | 1.47 [0.93–2.30]  -Reference- | 0.095 | 1.49 [0.95 – 2.34]  -Reference- | 0.079 |
| **General Health**  Good  Fair/Poor  Excellent | 1.04 [0.85 – 1.26]  1.03 [0.77 – 1.37]  -Reference- | 0.67  0.80 | 1.06 [0.84 – 1.35]  1.17 [0.81 – 1.68]  -Reference- | 0.594  0.381 | 1.07 [0.84 – 1.36]  1.19 [0.82 – 1.71]  -Reference- | 0.555  0.349 |
| **Physical activity ^ff^**  Low activity  High activity | 0.87 [0.73 – 1.03]  -Reference- | 0.12 | 1.10 [0.88 – 1.38]  -Reference- | 0.359 | 1.12 [0.90 – 1.40]  -Reference- | 0.283 |
| **Smoking status ^gg^**  Smoker  Non-Smoker | 1.21 [0.97 – 1.51]  -Reference- | 0.08 | 1.09 [0.83 –1.44]  -Reference- | 0.503 | 1.10 [0.84 – 1.45]  -Reference- | 0.473 |
| **Fruit/ vegetable consumption ^hh^**  Low intake  High intake | 1.78 [1.49 – 2.13]  -Reference- | <0.001 | 1.37 [1.09 – 1.71]  -Reference- | 0.006 | 1.37 [1.09 – 1.71]  -Reference- | 0.006 |
| **Bariatric surgery**  Yes  No | 1.38 [1.06 – 1.78]  -Reference- | 0.013 | 1.17 [0.85 – 1.61]  -Reference- | 0.317 | 1.19 [0.86 – 1.64]  -Reference- | 0.273 |
| **Shift work**  **Yes**  **NO** | 1.31[1.07 – 1.61]  -Reference- | 0.008 | 1.41 [1.09-1.82]  -Reference- | 0.008 | 1.40 [1.09 – 1.80]  -Reference- | 0.008 |
| **Income range (per month) ^gg^**  **>50,000 QAR**  **20,000-50,000QAR**  **>20,000 QAR** | 0.77 [1.06 – 1.59]  1.30 [1.06 – 1.59]  -Reference- | 0.062  0.009 | 1.01 [0.72 – 1.43]  1.17 [0.899-1.52]  -Reference- | 0.927  0.240 | 0.98 [0.69 – 1.39]  1.18 [0.91 – 1.54]  -Reference- | 0.940  0.208 |
| **Age (years) x Gender Interaction**  18–24 x Female  25–34 x Female  35–44 x Female  45-65 x Female |  |  |  |  | 3.06 [1.33 – 7.04]  1.73 [0.95 – 3.13]  1.87 [1.06 – 3.31]  Reference- | 0.008  0.07  0.03 |
| ***Footnote. Table S5***. Sensitivity Analysis: Subgroup Series Logistic Regression  The table presents the results from the sensitivity analysis performed on a subgroup of the study population consisting of employed participants with complete information about income and shift work status (employment status) (N = 1,660).  Results are presented across Crude, adjusted (main effects), and Final Interaction Models using purposeful selection and interaction testing procedures. Regular fast-food consumption was the dependent variable, and irregular consumption (< 1 time/week) served as the reference outcome.  Statistically significant association set at p <0.05; OR: Odds Ratio; AOR: Adjusted Odds Ratio, CI: Confident Interval.  aa Non-Qatari: including Arabs and other nationalities  bb Based on area zone, the urban from 1 to 69, while rural 70 to 98  cc Participants were categorized into less than high school level (did not attend school, did not complete primary school, primary school, secondary school), for high school level and higher (high school, Technical/ professional school, university degree, and postgraduate degree).  ee >25 kg/m^2^BMI (Underweight (<18.5), Normal weight (≥18.5 and <25.0)) while >25kg/m (Overweight (≥25.0 - <30.0), and Obese (≥30.0))  ff Participants were categorized into low leisure time physical activity (<600 total MET-minutes/week) and high leisure time physical activity (≥600 total MET-minutes/week).  gg Nonsmoker (No, have never smoked, no, stopped smoking, no, just have tried once or twice) and smoker (Yes, only occasionally, yes, on most or all days)  hh Median intake of fruit and vegetables was used as a cutoff point between high vs low intake.  ii Participants were categorized into unmarried (separated, single, widow) and married (married) | | | | | | |
